# Supplementary material for: Discovery and application of insertion-deletion (INDEL) polymorphisms for QTL mapping of early life-history traits in Atlantic salmon
Source: BMC Genomics. 2010 Mar 8;11:156. doi: 10.1186/1471-2164-11-156 (PMC2838853; doi:10.1186/1471-2164-11-156)
Supplement: Additional file 2 — Information on developed 76 locus single-run INDEL panel in Atlantic salmon. Information on fluorescence labeling, primer concentrations, PCR pooling and links to alignments, INDEL motifs and GENESCAN (Burge and Karlin 1997) predictions of genes/exons are available in html format. [file 1471-2164-11-156-S2.ZIP › Additionalfile2/snpsummary86.html]

```
Cluster 7 Contig 3

prev  Summary    Contig List  next
```

Size of Consensus sequence = 1686

Number of sequences = 172

Minimum redundancy = 6

Key

A gi|84208237|gb|DW179939.1|DW179939 SGP303546 Atlantic salmon Gills II cDNA library Salmo salar cDNA clone SS0103G02 5', mRNA sequence  
B gi|84210339|gb|DW180972.1|DW180972 SGP306909 Atlantic salmon Heart II cDNA library Salmo salar cDNA clone SS0051A11 5', mRNA sequence  
C gi|84209043|gb|DW180344.1|DW180344 SGP303972 Atlantic salmon Gills II cDNA library Salmo salar cDNA clone SS0196H03 5', mRNA sequence  
D gi|45314361|gb|CK884730.1|CK884730 SGP165090 Atlantic salmon Intestine cDNA library Salmo salar cDNA clone T4-0373 5', mRNA sequence  
E gi|117867628|gb|EG940324.1|EG940324 EST\_ssal\_evf\_23860 ssalevf mixed\_tissue Salmo salar cDNA Salmo salar cDNA clone ssal\_evf\_531\_201\_fwd 3', mRNA sequence  
F gi|84210955|gb|DW181284.1|DW181284 SGP307229 Atlantic salmon Heart II cDNA library Salmo salar cDNA clone SS0091C09 5', mRNA sequence  
G gi|45320505|gb|CK890772.1|CK890772 SGP163031 Atlantic salmon Ovaries cDNA library Salmo salar cDNA clone KG4-0270 5', mRNA sequence  
H gi|84981912|gb|DW532262.1|DW532262 EST\_ssal\_plnb\_898 plnb Salmo salar cDNA clone ssal\_plnb\_008\_038\_rev 5', mRNA sequence  
I gi|60104000|gb|DN163607.1|DN163607 SGP270072 Atlantic salmon White muscle cDNA library Salmo salar cDNA clone HM4-3089 5', mRNA sequence  
J gi|40541763|gb|BM414025.1|BM414025 EST00947 Atlantic salmon Lambda Zap Express ovary cDNA library Salmo salar cDNA clone oyr\_003\_c02 5' similar to Myosin regulatory light chain 2, P24032, mRNA sequence  
K gi|59834761|gb|DN140444.1|DN140444 SGP266478 Atlantic salmon Head kidney cDNA library Salmo salar cDNA clone FN4-3876 5', mRNA sequence  
L gi|59833248|gb|DN138931.1|DN138931 SGP263753 Atlantic salmon Head kidney cDNA library Salmo salar cDNA clone FN4-1922 5', mRNA sequence  
M gi|29314084|gb|CB502858.1|CB502858 ssalplnb510016\_rev gut Salmo salar cDNA, mRNA sequence  
N gi|45319197|gb|CK889464.1|CK889464 SGP161487 Atlantic salmon Liver cDNA library Salmo salar cDNA clone L4-1163 5', mRNA sequence  
O gi|117298288|gb|EG648101.1|EG648101 SGP261402 Atlantic salmon normalized Brain cDNA library Salmo salar cDNA clone NB1-1441 5', mRNA sequence  
P gi|15845654|gb|BG935826.1|BG935826 SS1-0142 Atlantic Salmon spleen Salmo salar cDNA clone SS1-0142 similar to Myosin regulatory light chain, mRNA sequence  
Q gi|45305242|gb|CK875611.1|CK875611 SGP136853 Atlantic salmon Eye cDNA library Salmo salar cDNA clone OY6-0408 5', mRNA sequence  
R gi|84971384|gb|DW469785.1|DW469785 SGP298664 Atlantic salmon Testis cDNA library Salmo salar cDNA clone MG4-2961 5', mRNA sequence  
S gi|117485523|gb|EG817740.1|EG817740 EST\_ssal\_evd\_19946 ssalevd thymus Salmo salar cDNA Salmo salar cDNA clone ssal\_evd\_525\_256\_fwd 3', mRNA sequence  
T gi|57122390|gb|CX353831.1|CX353831 ssalrgb514083\_rev\_0 mixed\_tissue Salmo salar cDNA, mRNA sequence  
U gi|29314455|gb|CB503229.1|CB503229 ssalplnb510165\_rev gut Salmo salar cDNA, mRNA sequence  
V gi|50135123|gb|CO469924.1|CO469924 SGP169849 Atlantic salmon Eye cDNA library Salmo salar cDNA clone OY4-1315 5', mRNA sequence  
W gi|45324647|gb|CK894914.1|CK894914 SGP160528 Atlantic salmon Spleen cDNA library Salmo salar cDNA clone MI5-1244 5', mRNA sequence  
X gi|117517947|gb|EG849706.1|EG849706 EST\_ssal\_eve\_7127 ssaleve thyroid Salmo salar cDNA Salmo salar cDNA clone ssal\_eve\_508\_256\_fwd 3', mRNA sequence  
Y gi|117849227|gb|EG921923.1|EG921923 EST\_ssal\_evf\_1087 ssalevf mixed\_tissue Salmo salar cDNA Salmo salar cDNA clone ssal\_evf\_005\_216\_fwd 3', mRNA sequence  
Z gi|117532238|gb|EG863683.1|EG863683 EST\_ssal\_eve\_56607 ssaleve thyroid Salmo salar cDNA Salmo salar cDNA clone ssal\_eve\_577\_054\_fwd 3', mRNA sequence  
a gi|117570064|gb|EG891040.1|EG891040 EST\_ssal\_evf\_48837 ssalevf mixed\_tissue Salmo salar cDNA Salmo salar cDNA clone ssal\_evf\_565\_127\_fwd 3', mRNA sequence  
b gi|117519189|gb|EG850917.1|EG850917 EST\_ssal\_eve\_8218 ssaleve thyroid Salmo salar cDNA Salmo salar cDNA clone ssal\_eve\_510\_048\_fwd 3', mRNA sequence  
c gi|117498256|gb|EG830473.1|EG830473 EST\_ssal\_eve\_42719 ssaleve thyroid Salmo salar cDNA Salmo salar cDNA clone ssal\_eve\_558\_039\_fwd 3', mRNA sequence  
d gi|117453774|gb|EG785993.1|EG785993 EST\_ssal\_evd\_6712 ssalevd thymus Salmo salar cDNA Salmo salar cDNA clone ssal\_evd\_507\_261\_fwd 3', mRNA sequence  
e gi|84973043|gb|DW471444.1|DW471444 SGP285561 Atlantic salmon Spleen cDNA library Salmo salar cDNA clone MI5-1340 5', mRNA sequence  
f gi|117548282|gb|EG879727.1|EG879727 EST\_ssal\_eve\_23115 ssaleve thyroid Salmo salar cDNA Salmo salar cDNA clone ssal\_eve\_531\_187\_fwd 3', mRNA sequence  
g gi|117507933|gb|EG839692.1|EG839692 EST\_ssal\_eve\_2617 ssaleve thyroid Salmo salar cDNA Salmo salar cDNA clone ssal\_eve\_502\_231\_fwd 3', mRNA sequence  
h gi|117521485|gb|EG853212.1|EG853212 EST\_ssal\_eve\_14083 ssaleve thyroid Salmo salar cDNA Salmo salar cDNA clone ssal\_eve\_519\_051\_fwd 3', mRNA sequence  
i gi|117536375|gb|EG867820.1|EG867820 EST\_ssal\_eve\_18151 ssaleve thyroid Salmo salar cDNA Salmo salar cDNA clone ssal\_eve\_524\_285\_fwd 3', mRNA sequence  
j gi|117462711|gb|EG794930.1|EG794930 EST\_ssal\_evd\_14757 ssalevd thymus Salmo salar cDNA Salmo salar cDNA clone ssal\_evd\_518\_246\_fwd 3', mRNA sequence  
k gi|117501428|gb|EG833444.1|EG833444 EST\_ssal\_eve\_45392 ssaleve thyroid Salmo salar cDNA Salmo salar cDNA clone ssal\_eve\_561\_286\_fwd 3', mRNA sequence  
l gi|117832849|gb|EG905545.1|EG905545 EST\_ssal\_evf\_8147 ssalevf mixed\_tissue Salmo salar cDNA Salmo salar cDNA clone ssal\_evf\_509\_092\_fwd 3', mRNA sequence  
m gi|117437918|gb|EG770141.1|EG770141 EST\_ssal\_evd\_45911 ssalevd thymus Salmo salar cDNA Salmo salar cDNA clone ssal\_evd\_561\_254\_fwd 3', mRNA sequence  
n gi|85055262|gb|DW583440.1|DW583440 EST\_ssal\_rgb2\_47859 rgb2 Salmo salar cDNA clone ssal\_rgb2\_577\_110\_rev 5', mRNA sequence  
o gi|117841165|gb|EG913861.1|EG913861 EST\_ssal\_evf\_15633 ssalevf mixed\_tissue Salmo salar cDNA Salmo salar cDNA clone ssal\_evf\_519\_162\_fwd 3', mRNA sequence  
p gi|117845993|gb|EG918689.1|EG918689 EST\_ssal\_evf\_57717 ssalevf mixed\_tissue Salmo salar cDNA Salmo salar cDNA clone ssal\_evf\_577\_171\_fwd 3', mRNA sequence  
q gi|85015778|gb|DW544434.1|DW544434 EST\_ssal\_rgb2\_8853 rgb2 Salmo salar cDNA clone ssal\_rgb2\_515\_125\_rev 5', mRNA sequence  
r gi|89840259|gb|DY698182.1|DY698182 EST\_ssal\_rgb2\_53921 ssalrgb2 mixed\_tissue Salmo salar cDNA Salmo salar cDNA clone ssal\_rgb2\_587\_074\_rev 5', mRNA sequence  
s gi|117854613|gb|EG927309.1|EG927309 EST\_ssal\_evf\_18017 ssalevf mixed\_tissue Salmo salar cDNA Salmo salar cDNA clone ssal\_evf\_522\_239\_fwd 3', mRNA sequence  
t gi|117490324|gb|EG822541.1|EG822541 EST\_ssal\_evd\_24268 ssalevd thymus Salmo salar cDNA Salmo salar cDNA clone ssal\_evd\_531\_206\_fwd 3', mRNA sequence  
u gi|117506898|gb|EG838657.1|EG838657 EST\_ssal\_eve\_1683 ssaleve thyroid Salmo salar cDNA Salmo salar cDNA clone ssal\_eve\_501\_136\_fwd 3', mRNA sequence  
v gi|117504107|gb|EG835866.1|EG835866 EST\_ssal\_eve\_47572 ssaleve thyroid Salmo salar cDNA Salmo salar cDNA clone ssal\_eve\_564\_265\_fwd 3', mRNA sequence  
w gi|89828866|gb|DY691792.1|DY691792 EST\_ssal\_plnb\_4702 ssalplnb mixed\_tissue Salmo salar cDNA Salmo salar cDNA clone ssal\_plnb\_024\_353\_rev 5', mRNA sequence  
x gi|45314788|gb|CK885157.1|CK885157 SGP165524 Atlantic salmon Intestine cDNA library Salmo salar cDNA clone T4-0440 5', mRNA sequence  
y gi|117501259|gb|EG833277.1|EG833277 EST\_ssal\_eve\_45241 ssaleve thyroid Salmo salar cDNA Salmo salar cDNA clone ssal\_eve\_561\_205\_fwd 3', mRNA sequence  
z gi|89850884|gb|DY707007.1|DY707007 EST\_ssal\_rgb2\_62746 ssalrgb2 mixed\_tissue Salmo salar cDNA Salmo salar cDNA clone ssal\_rgb2\_601\_131\_rev 5', mRNA sequence  
A gi|117450845|gb|EG783064.1|EG783064 EST\_ssal\_evd\_39542 ssalevd thymus Salmo salar cDNA Salmo salar cDNA clone ssal\_evd\_552\_351\_fwd 3', mRNA sequence  
B gi|84568067|gb|DW339686.1|DW339686 SGP302448 Atlantic salmon Ovaries cDNA library Salmo salar cDNA clone KG4-2756 5', mRNA sequence  
C gi|117507699|gb|EG839458.1|EG839458 EST\_ssal\_eve\_2406 ssaleve thyroid Salmo salar cDNA Salmo salar cDNA clone ssal\_eve\_502\_123\_fwd 3', mRNA sequence  
D gi|117840417|gb|EG913113.1|EG913113 EST\_ssal\_evf\_14959 ssalevf mixed\_tissue Salmo salar cDNA Salmo salar cDNA clone ssal\_evf\_518\_199\_fwd 3', mRNA sequence  
E gi|117536648|gb|EG868093.1|EG868093 EST\_ssal\_eve\_29476 ssaleve thyroid Salmo salar cDNA Salmo salar cDNA clone ssal\_eve\_540\_014\_fwd 3', mRNA sequence  
F gi|45319528|gb|CK889795.1|CK889795 SGP150017 Atlantic salmon Ovaries cDNA library Salmo salar cDNA clone KG6-0331 5', mRNA sequence  
G gi|117853853|gb|EG926549.1|EG926549 EST\_ssal\_evf\_29051 ssalevf mixed\_tissue Salmo salar cDNA Salmo salar cDNA clone ssal\_evf\_538\_201\_fwd 3', mRNA sequence  
H gi|85158533|gb|DW589835.1|DW589835 sbra1-012AF12.g1\_#0028.0 DIAS\_SBRA Salmo salar cDNA 5', mRNA sequence  
I gi|84970632|gb|DW469033.1|DW469033 SGP286914 Atlantic salmon Testis cDNA library Salmo salar cDNA clone MG4-2060 5', mRNA sequence  
J gi|117453095|gb|EG785314.1|EG785314 EST\_ssal\_evd\_41567 ssalevd thymus Salmo salar cDNA Salmo salar cDNA clone ssal\_evd\_555\_264\_fwd 3', mRNA sequence  
K gi|84974264|gb|DW472665.1|DW472665 SGP301311 Atlantic salmon Spleen cDNA library Salmo salar cDNA clone MI5-2714 5', mRNA sequence  
L gi|117837266|gb|EG909962.1|EG909962 EST\_ssal\_evf\_12123 ssalevf mixed\_tissue Salmo salar cDNA Salmo salar cDNA clone ssal\_evf\_514\_263\_fwd 3', mRNA sequence  
M gi|117506899|gb|EG838658.1|EG838658 EST\_ssal\_eve\_1684 ssaleve thyroid Salmo salar cDNA Salmo salar cDNA clone ssal\_eve\_501\_136\_rev 5', mRNA sequence  
N gi|117850118|gb|EG922814.1|EG922814 EST\_ssal\_evf\_1890 ssalevf mixed\_tissue Salmo salar cDNA Salmo salar cDNA clone ssal\_evf\_006\_271\_fwd 3', mRNA sequence  
O gi|117536649|gb|EG868094.1|EG868094 EST\_ssal\_eve\_29477 ssaleve thyroid Salmo salar cDNA Salmo salar cDNA clone ssal\_eve\_540\_014\_rev 5', mRNA sequence  
P gi|89764954|gb|DY691539.1|DY691539 EST\_ssal\_plnb\_4449 ssalplnb mixed\_tissue Salmo salar cDNA Salmo salar cDNA clone ssal\_plnb\_024\_169\_rev 5', mRNA sequence  
Q gi|117866225|gb|EG938921.1|EG938921 EST\_ssal\_evf\_22598 ssalevf mixed\_tissue Salmo salar cDNA Salmo salar cDNA clone ssal\_evf\_529\_290\_fwd 3', mRNA sequence  
R gi|117570062|gb|EG891038.1|EG891038 EST\_ssal\_evf\_48836 ssalevf mixed\_tissue Salmo salar cDNA Salmo salar cDNA clone ssal\_evf\_565\_127\_rev 5', mRNA sequence  
S gi|117501427|gb|EG833443.1|EG833443 EST\_ssal\_eve\_45391 ssaleve thyroid Salmo salar cDNA Salmo salar cDNA clone ssal\_eve\_561\_286\_rev 5', mRNA sequence  
T gi|117528449|gb|EG860090.1|EG860090 EST\_ssal\_eve\_53372 ssaleve thyroid Salmo salar cDNA Salmo salar cDNA clone ssal\_eve\_572\_280\_fwd 3', mRNA sequence  
U gi|75981038|gb|AM041669.1|AM041669 AM041669 pGemT-easy Atlantic salmon gill subtracted library (Aeromonas salmonicda infected salmon subtracted with uninfected) Salmo salar cDNA clone GT4B4, mRNA sequence  
V gi|50140565|gb|CO472438.1|CO472438 SGP214385 Atlantic salmon Eye cDNA library Salmo salar cDNA clone OY5-0681 5', mRNA sequence  
W gi|59636333|gb|DN047571.1|DN047571 ssallksd011035\_0 mixed\_tissue\_SDb Salmo salar cDNA, mRNA sequence  
X gi|24343700|gb|CA042780.1|CA042780 ssalplnb504142 gut Salmo salar cDNA, mRNA sequence  
Y gi|117478572|gb|EG810791.1|EG810791 EST\_ssal\_evd\_31031 ssalevd thymus Salmo salar cDNA Salmo salar cDNA clone ssal\_evd\_540\_373\_fwd 3', mRNA sequence  
Z gi|45307545|gb|CK877914.1|CK877914 SGP139259 Atlantic salmon Gills cDNA library Salmo salar cDNA clone G6-1372 5', mRNA sequence  
a gi|117456901|gb|EG789120.1|EG789120 EST\_ssal\_evd\_9528 ssalevd thymus Salmo salar cDNA Salmo salar cDNA clone ssal\_evd\_511\_194\_fwd 3', mRNA sequence  
b gi|117446780|gb|EG778999.1|EG778999 EST\_ssal\_evd\_5483 ssalevd thymus Salmo salar cDNA Salmo salar cDNA clone ssal\_evd\_506\_001\_fwd 3', mRNA sequence  
c gi|117866017|gb|EG938713.1|EG938713 EST\_ssal\_evf\_22411 ssalevf mixed\_tissue Salmo salar cDNA Salmo salar cDNA clone ssal\_evf\_529\_194\_fwd 3', mRNA sequence  
d gi|24336037|gb|CA036920.1|CA036920 ssalob009084 reproductive Salmo salar cDNA, mRNA sequence  
e gi|25997256|gb|CA768001.1|CA768001 ssalplnb507335 gut Salmo salar cDNA, mRNA sequence  
f gi|84981913|gb|DW532263.1|DW532263 EST\_ssal\_plnb\_899 plnb Salmo salar cDNA clone ssal\_plnb\_008\_038\_fwd 3', mRNA sequence  
g gi|24344657|gb|CA043737.1|CA043737 ssalplnb510165 gut Salmo salar cDNA, mRNA sequence  
h gi|117510130|gb|EG841889.1|EG841889 EST\_ssal\_eve\_4594 ssaleve thyroid Salmo salar cDNA Salmo salar cDNA clone ssal\_eve\_505\_097\_fwd 3', mRNA sequence  
i gi|50137351|gb|CO470955.1|CO470955 SGP212681 Atlantic salmon Eye cDNA library Salmo salar cDNA clone OY4-2540 5', mRNA sequence  
j gi|117477363|gb|EG809582.1|EG809582 EST\_ssal\_evd\_29943 ssalevd thymus Salmo salar cDNA Salmo salar cDNA clone ssal\_evd\_539\_114\_fwd 3', mRNA sequence  
k gi|117866224|gb|EG938920.1|EG938920 EST\_ssal\_evf\_22597 ssalevf mixed\_tissue Salmo salar cDNA Salmo salar cDNA clone ssal\_evf\_529\_290\_rev 5', mRNA sequence  
l gi|117564680|gb|EG885656.1|EG885656 EST\_ssal\_evf\_43991 ssalevf mixed\_tissue Salmo salar cDNA Salmo salar cDNA clone ssal\_evf\_558\_304\_fwd 3', mRNA sequence  
m gi|117435965|gb|EG768188.1|EG768188 EST\_ssal\_evd\_44154 ssalevd thymus Salmo salar cDNA Salmo salar cDNA clone ssal\_evd\_559\_103\_fwd 3', mRNA sequence  
n gi|117515878|gb|EG847637.1|EG847637 EST\_ssal\_eve\_5265 ssaleve thyroid Salmo salar cDNA Salmo salar cDNA clone ssal\_eve\_506\_065\_fwd 3', mRNA sequence  
o gi|117843749|gb|EG916445.1|EG916445 EST\_ssal\_evf\_55697 ssalevf mixed\_tissue Salmo salar cDNA Salmo salar cDNA clone ssal\_evf\_574\_262\_fwd 3', mRNA sequence  
p gi|117446779|gb|EG778998.1|EG778998 EST\_ssal\_evd\_5482 ssalevd thymus Salmo salar cDNA Salmo salar cDNA clone ssal\_evd\_506\_001\_rev 5', mRNA sequence  
q gi|45324028|gb|CK894295.1|CK894295 SGP159900 Atlantic salmon Spleen cDNA library Salmo salar cDNA clone MI5-0316 5', mRNA sequence  
r gi|117504790|gb|EG836549.1|EG836549 EST\_ssal\_eve\_11185 ssaleve thyroid Salmo salar cDNA Salmo salar cDNA clone ssal\_eve\_515\_072\_fwd 3', mRNA sequence  
s gi|24355220|gb|CA049050.1|CA049050 ssalkspa003053 kidney Salmo salar cDNA, mRNA sequence  
t gi|70786318|gb|DR695958.1|DR695958 SMT6-0137 Atlantic Salmon macrophage - Aeromonas in vitro infection Salmo salar cDNA clone SMT6-0137, mRNA sequence  
u gi|75980971|gb|AM041602.1|AM041602 AM041602 pGemT-easy Atlantic salmon gill subtracted library (Aeromonas salmonicda infected salmon subtracted with uninfected) Salmo salar cDNA clone GT3D1, mRNA sequence  
v gi|117488761|gb|EG820978.1|EG820978 EST\_ssal\_evd\_22860 ssalevd thymus Salmo salar cDNA Salmo salar cDNA clone ssal\_evd\_529\_235\_fwd 3', mRNA sequence  
w gi|117860951|gb|EG933647.1|EG933647 EST\_ssal\_evf\_35440 ssalevf mixed\_tissue Salmo salar cDNA Salmo salar cDNA clone ssal\_evf\_547\_065\_fwd 3', mRNA sequence  
x gi|117846406|gb|EG919102.1|EG919102 EST\_ssal\_evf\_58089 ssalevf mixed\_tissue Salmo salar cDNA Salmo salar cDNA clone ssal\_evf\_578\_005\_fwd 3', mRNA sequence  
y gi|117435964|gb|EG768187.1|EG768187 EST\_ssal\_evd\_44153 ssalevd thymus Salmo salar cDNA Salmo salar cDNA clone ssal\_evd\_559\_101\_fwd 3', mRNA sequence  
z gi|117490325|gb|EG822542.1|EG822542 EST\_ssal\_evd\_24269 ssalevd thymus Salmo salar cDNA Salmo salar cDNA clone ssal\_evd\_531\_206\_rev 5', mRNA sequence  
A gi|117507700|gb|EG839459.1|EG839459 EST\_ssal\_eve\_2407 ssaleve thyroid Salmo salar cDNA Salmo salar cDNA clone ssal\_eve\_502\_123\_rev 5', mRNA sequence  
B gi|117507934|gb|EG839693.1|EG839693 EST\_ssal\_eve\_2618 ssaleve thyroid Salmo salar cDNA Salmo salar cDNA clone ssal\_eve\_502\_231\_rev 5', mRNA sequence  
C gi|117453773|gb|EG785992.1|EG785992 EST\_ssal\_evd\_6711 ssalevd thymus Salmo salar cDNA Salmo salar cDNA clone ssal\_evd\_507\_261\_rev 5', mRNA sequence  
D gi|117435967|gb|EG768190.1|EG768190 EST\_ssal\_evd\_44155 ssalevd thymus Salmo salar cDNA Salmo salar cDNA clone ssal\_evd\_559\_103\_rev 5', mRNA sequence  
E gi|117450844|gb|EG783063.1|EG783063 EST\_ssal\_evd\_39541 ssalevd thymus Salmo salar cDNA Salmo salar cDNA clone ssal\_evd\_552\_351\_rev 5', mRNA sequence  
F gi|29311768|gb|CB500542.1|CB500542 ssalga503193 head Salmo salar cDNA, mRNA sequence  
G gi|117511670|gb|EG843429.1|EG843429 EST\_ssal\_eve\_36380 ssaleve thyroid Salmo salar cDNA Salmo salar cDNA clone ssal\_eve\_549\_190\_fwd 3', mRNA sequence  
H gi|29317203|gb|CB505977.1|CB505977 ssalmge502044 gut Salmo salar cDNA, mRNA sequence  
I gi|89764953|gb|DY691538.1|DY691538 EST\_ssal\_plnb\_4448 ssalplnb mixed\_tissue Salmo salar cDNA Salmo salar cDNA clone ssal\_plnb\_024\_169\_fwd 3', mRNA sequence  
J gi|25997668|gb|CA768413.1|CA768413 ssalga007033 head Salmo salar cDNA, mRNA sequence  
K gi|24394391|gb|CA064148.1|CA064148 ssalrgb514083 mixed\_tissue Salmo salar cDNA, mRNA sequence  
L gi|24344556|gb|CA043636.1|CA043636 ssalplnb510016 gut Salmo salar cDNA, mRNA sequence  
M gi|117853852|gb|EG926548.1|EG926548 EST\_ssal\_evf\_29050 ssalevf mixed\_tissue Salmo salar cDNA Salmo salar cDNA clone ssal\_evf\_538\_201\_rev 5', mRNA sequence  
N gi|29312564|gb|CB501338.1|CB501338 ssalga501379 head Salmo salar cDNA, mRNA sequence  
O gi|117477364|gb|EG809583.1|EG809583 EST\_ssal\_evd\_29944 ssalevd thymus Salmo salar cDNA Salmo salar cDNA clone ssal\_evd\_539\_114\_rev 5', mRNA sequence  
P gi|117843750|gb|EG916446.1|EG916446 EST\_ssal\_evf\_55698 ssalevf mixed\_tissue Salmo salar cDNA Salmo salar cDNA clone ssal\_evf\_574\_262\_rev 5', mRNA sequence  
Q gi|117860952|gb|EG933648.1|EG933648 EST\_ssal\_evf\_35441 ssalevf mixed\_tissue Salmo salar cDNA Salmo salar cDNA clone ssal\_evf\_547\_065\_rev 5', mRNA sequence  
R gi|117845992|gb|EG918688.1|EG918688 EST\_ssal\_evf\_57716 ssalevf mixed\_tissue Salmo salar cDNA Salmo salar cDNA clone ssal\_evf\_577\_171\_rev 5', mRNA sequence  
S gi|117521484|gb|EG853211.1|EG853211 EST\_ssal\_eve\_14082 ssaleve thyroid Salmo salar cDNA Salmo salar cDNA clone ssal\_eve\_519\_051\_rev 5', mRNA sequence  
T gi|117532239|gb|EG863684.1|EG863684 EST\_ssal\_eve\_56608 ssaleve thyroid Salmo salar cDNA Salmo salar cDNA clone ssal\_eve\_577\_054\_rev 5', mRNA sequence  
U gi|117519188|gb|EG850916.1|EG850916 EST\_ssal\_eve\_8217 ssaleve thyroid Salmo salar cDNA Salmo salar cDNA clone ssal\_eve\_510\_048\_rev 5', mRNA sequence  
V gi|117504791|gb|EG836550.1|EG836550 EST\_ssal\_eve\_11186 ssaleve thyroid Salmo salar cDNA Salmo salar cDNA clone ssal\_eve\_515\_072\_rev 5', mRNA sequence  
W gi|117832850|gb|EG905546.1|EG905546 EST\_ssal\_evf\_8148 ssalevf mixed\_tissue Salmo salar cDNA Salmo salar cDNA clone ssal\_evf\_509\_092\_rev 5', mRNA sequence  
X gi|117462712|gb|EG794931.1|EG794931 EST\_ssal\_evd\_14758 ssalevd thymus Salmo salar cDNA Salmo salar cDNA clone ssal\_evd\_518\_246\_rev 5', mRNA sequence  
Y gi|117536386|gb|EG867831.1|EG867831 EST\_ssal\_eve\_18152 ssaleve thyroid Salmo salar cDNA Salmo salar cDNA clone ssal\_eve\_524\_285\_rev 5', mRNA sequence  
Z gi|117866016|gb|EG938712.1|EG938712 EST\_ssal\_evf\_22410 ssalevf mixed\_tissue Salmo salar cDNA Salmo salar cDNA clone ssal\_evf\_529\_194\_rev 5', mRNA sequence  
a gi|117515879|gb|EG847638.1|EG847638 EST\_ssal\_eve\_5266 ssaleve thyroid Salmo salar cDNA Salmo salar cDNA clone ssal\_eve\_506\_065\_rev 5', mRNA sequence  
b gi|117837265|gb|EG909961.1|EG909961 EST\_ssal\_evf\_12122 ssalevf mixed\_tissue Salmo salar cDNA Salmo salar cDNA clone ssal\_evf\_514\_263\_rev 5', mRNA sequence  
c gi|117564679|gb|EG885655.1|EG885655 EST\_ssal\_evf\_43990 ssalevf mixed\_tissue Salmo salar cDNA Salmo salar cDNA clone ssal\_evf\_558\_304\_rev 5', mRNA sequence  
d gi|117528448|gb|EG860089.1|EG860089 EST\_ssal\_eve\_53371 ssaleve thyroid Salmo salar cDNA Salmo salar cDNA clone ssal\_eve\_572\_280\_rev 5', mRNA sequence  
e gi|45312188|gb|CK882557.1|CK882557 SGP147192 Atlantic salmon Heart cDNA library Salmo salar cDNA clone H7-0478 5', mRNA sequence  
f gi|29320189|gb|CB508671.1|CB508671 ssaltc020002 reproductive Salmo salar cDNA, mRNA sequence  
g gi|45324752|gb|CK895019.1|CK895019 SGP154265 Atlantic salmon Swimbladder cDNA library Salmo salar cDNA clone SB7-0170 5', mRNA sequence  
h gi|117467612|gb|EG799831.1|EG799831 EST\_ssal\_evd\_56631 ssalevd thymus Salmo salar cDNA Salmo salar cDNA clone ssal\_evd\_576\_144\_rev 5', mRNA sequence  
i gi|117467613|gb|EG799832.1|EG799832 EST\_ssal\_evd\_56632 ssalevd thymus Salmo salar cDNA Salmo salar cDNA clone ssal\_evd\_576\_144\_fwd 3', mRNA sequence  
j gi|117479661|gb|EG811878.1|EG811878 EST\_ssal\_evd\_18240 ssalevd thymus Salmo salar cDNA Salmo salar cDNA clone ssal\_evd\_523\_144\_rev 5', mRNA sequence  
k gi|117479672|gb|EG811889.1|EG811889 EST\_ssal\_evd\_18241 ssalevd thymus Salmo salar cDNA Salmo salar cDNA clone ssal\_evd\_523\_144\_fwd 3', mRNA sequence  
l gi|117451424|gb|EG783643.1|EG783643 EST\_ssal\_evd\_40064 ssalevd thymus Salmo salar cDNA Salmo salar cDNA clone ssal\_evd\_553\_244\_rev 5', mRNA sequence  
m gi|117451426|gb|EG783645.1|EG783645 EST\_ssal\_evd\_40065 ssalevd thymus Salmo salar cDNA Salmo salar cDNA clone ssal\_evd\_553\_244\_fwd 3', mRNA sequence  
n gi|89850885|gb|DY707008.1|DY707008 EST\_ssal\_rgb2\_62747 ssalrgb2 mixed\_tissue Salmo salar cDNA Salmo salar cDNA clone ssal\_rgb2\_601\_131\_fwd 3', mRNA sequence  
o gi|89840257|gb|DY698181.1|DY698181 EST\_ssal\_rgb2\_53920 ssalrgb2 mixed\_tissue Salmo salar cDNA Salmo salar cDNA clone ssal\_rgb2\_587\_074\_fwd 3', mRNA sequence  
p gi|45316709|gb|CK887078.1|CK887078 SGP148983 Atlantic salmon Kidney cDNA library Salmo salar cDNA clone BN6-0543 5', mRNA sequence  
q gi|29318058|gb|CB509347.1|CB509347 ssalmna005043 muscle\_skin Salmo salar cDNA, mRNA sequence  
r gi|117456902|gb|EG789121.1|EG789121 EST\_ssal\_evd\_9529 ssalevd thymus Salmo salar cDNA Salmo salar cDNA clone ssal\_evd\_511\_194\_rev 5', mRNA sequence  
s gi|117548281|gb|EG879726.1|EG879726 EST\_ssal\_eve\_23114 ssaleve thyroid Salmo salar cDNA Salmo salar cDNA clone ssal\_eve\_531\_187\_rev 5', mRNA sequence  
t gi|117485522|gb|EG817739.1|EG817739 EST\_ssal\_evd\_19945 ssalevd thymus Salmo salar cDNA Salmo salar cDNA clone ssal\_evd\_525\_256\_rev 5', mRNA sequence  
u gi|117435963|gb|EG768186.1|EG768186 EST\_ssal\_evd\_44152 ssalevd thymus Salmo salar cDNA Salmo salar cDNA clone ssal\_evd\_559\_101\_rev 5', mRNA sequence  
v gi|117453096|gb|EG785315.1|EG785315 EST\_ssal\_evd\_41568 ssalevd thymus Salmo salar cDNA Salmo salar cDNA clone ssal\_evd\_555\_264\_rev 5', mRNA sequence  
w gi|117437919|gb|EG770142.1|EG770142 EST\_ssal\_evd\_45912 ssalevd thymus Salmo salar cDNA Salmo salar cDNA clone ssal\_evd\_561\_254\_rev 5', mRNA sequence  
x gi|117854602|gb|EG927298.1|EG927298 EST\_ssal\_evf\_18016 ssalevf mixed\_tissue Salmo salar cDNA Salmo salar cDNA clone ssal\_evf\_522\_239\_rev 5', mRNA sequence  
y gi|117488759|gb|EG820976.1|EG820976 EST\_ssal\_evd\_22859 ssalevd thymus Salmo salar cDNA Salmo salar cDNA clone ssal\_evd\_529\_235\_rev 5', mRNA sequence  
z gi|117465466|gb|EG797685.1|EG797685 EST\_ssal\_evd\_54698 ssalevd thymus Salmo salar cDNA Salmo salar cDNA clone ssal\_evd\_573\_276\_rev 5', mRNA sequence  
A gi|117465467|gb|EG797686.1|EG797686 EST\_ssal\_evd\_54699 ssalevd thymus Salmo salar cDNA Salmo salar cDNA clone ssal\_evd\_573\_276\_fwd 3', mRNA sequence  
B gi|117849226|gb|EG921922.1|EG921922 EST\_ssal\_evf\_1086 ssalevf mixed\_tissue Salmo salar cDNA Salmo salar cDNA clone ssal\_evf\_005\_216\_rev 5', mRNA sequence  
C gi|24342595|gb|CA041675.1|CA041675 ssalmge001088 gut Salmo salar cDNA, mRNA sequence  
D gi|117510129|gb|EG841888.1|EG841888 EST\_ssal\_eve\_4593 ssaleve thyroid Salmo salar cDNA Salmo salar cDNA clone ssal\_eve\_505\_097\_rev 5', mRNA sequence  
E gi|117841166|gb|EG913862.1|EG913862 EST\_ssal\_evf\_15634 ssalevf mixed\_tissue Salmo salar cDNA Salmo salar cDNA clone ssal\_evf\_519\_162\_rev 5', mRNA sequence  
F gi|117464487|gb|EG796706.1|EG796706 EST\_ssal\_evd\_16356 ssalevd thymus Salmo salar cDNA Salmo salar cDNA clone ssal\_evd\_520\_330\_rev 5', mRNA sequence  
G gi|117440878|gb|EG773101.1|EG773101 EST\_ssal\_evd\_3415 ssalevd thymus Salmo salar cDNA Salmo salar cDNA clone ssal\_evd\_503\_069\_fwd 3', mRNA sequence  
H gi|117440889|gb|EG773112.1|EG773112 EST\_ssal\_evd\_3416 ssalevd thymus Salmo salar cDNA Salmo salar cDNA clone ssal\_evd\_503\_069\_rev 5', mRNA sequence  
I gi|117478571|gb|EG810790.1|EG810790 EST\_ssal\_evd\_31030 ssalevd thymus Salmo salar cDNA Salmo salar cDNA clone ssal\_evd\_540\_373\_rev 5', mRNA sequence  
J gi|117464488|gb|EG796707.1|EG796707 EST\_ssal\_evd\_16357 ssalevd thymus Salmo salar cDNA Salmo salar cDNA clone ssal\_evd\_520\_330\_fwd 3', mRNA sequence  
K gi|117517946|gb|EG849705.1|EG849705 EST\_ssal\_eve\_7126 ssaleve thyroid Salmo salar cDNA Salmo salar cDNA clone ssal\_eve\_508\_256\_rev 5', mRNA sequence  
L gi|117511669|gb|EG843428.1|EG843428 EST\_ssal\_eve\_36379 ssaleve thyroid Salmo salar cDNA Salmo salar cDNA clone ssal\_eve\_549\_190\_rev 5', mRNA sequence  
M gi|117504108|gb|EG835867.1|EG835867 EST\_ssal\_eve\_47573 ssaleve thyroid Salmo salar cDNA Salmo salar cDNA clone ssal\_eve\_564\_265\_rev 5', mRNA sequence  
N gi|117456935|gb|EG789154.1|EG789154 EST\_ssal\_evd\_9559 ssalevd thymus Salmo salar cDNA Salmo salar cDNA clone ssal\_evd\_511\_212\_fwd 3', mRNA sequence  
O gi|84974857|gb|DW473258.1|DW473258 SGP314123 Atlantic salmon Spleen cDNA library Salmo salar cDNA clone MI5-3383 5', mRNA sequence  
P gi|89828868|gb|DY691793.1|DY691793 EST\_ssal\_plnb\_4703 ssalplnb mixed\_tissue Salmo salar cDNA Salmo salar cDNA clone ssal\_plnb\_024\_353\_fwd 3', mRNA sequence

5 SNPs detected

A B C D E F G H I J K L M N O P Q R S T U V W X Y Z a b c d e f g h i j k l m n o p q r s t u v w x y z A B C D E F G H I J K L M N O P Q R S T U V W X Y Z a b c d e f g h i j k l m n o p q r s t u v w x y z A B C D E F G H I J K L M N O P Q R S T U V W X Y Z a b c d e f g h i j k l m n o p q r s t u v w x y z A B C D E F G H I J K L M N O P  cosegregation weighted

72 A G G A A A . A G G A . A A . . . A A A A . . A A A A A A A . A A A A A A A A A A . A G A A A A . . G G A . A A A . . . . . . . . . . . . . . . . . . . . . . . . . . . . . . . . . . . . . . . . . . . . . . . . . . . . . . . . . . . . . . . . . . . . . . . . . . . . . . . . . . . . . . . . . . . . . . . . . . . . . . . . . . . . . . . . . . .   2/5 10.47
634 . . . . . . . . . . . . . . . A A A - - - A A A A A A A - - - A A A A A A - - - - - - A - A A A A A A A - - A - - - - A A - A - A - - A A A A - . A A A . . . - - . . . . A . C A - A . . . . . . . . . . . . . . . . . . . . . . . . . . . . . . . . . . . . . . . . . . . . . . . . . . . . . . . . . . . . . . . . . . . . . . . . . . . . . . . . .   1/5 7.79
635 . . . . . . . . . . . . . . . A A A - - - A A A A A A A - - - A A A A A A - - - - - - A - A A A A A A A - - A - - - - A A - A - A - - A A A A - . A A A . . . - - . . . . A . A A - A . . . . . . . . . . . . . . . . . . . . . . . . . . . . . . . . . . . . . . . . . . . . . . . . . . . . . . . . . . . . . . . . . . . . . . . . . . . . . . . . .   2/5 15.58
636 . . . . . . . . . . . . . . . T T T - - - T T T T T T T - - - T T T T T T - - - - - - T - T T T T T T T - - T - - - - T T - T - T - - T T T T - . T T T . . . - - . . . . T . T T - T . . . . . . . . . . . . . . . . . . . . . . . . . . . . . . . . . . . . . . . . . . . . . . . . . . . . . . . . . . . . . . . . . . . . . . . . . . . . . . . . .   2/5 15.58
1442 . . . . . . . . . . . . . . . . . . . . . . . . . . . . . . . . . . . . . . . . . . . . . . . . . . . . . . . . . . . . . . . . . . . . . . . . . . . . . . . . . . . . . . . . . . . . . . . T - T T T T T - T T T T - T T T T T T T T T T T T T T T T T T T T T T T T T T . . . T T T T T T - - T T T T T - T T T T T T T T T T T T T T T T T T T T T   2/5 17.21
